# Discontinuation of cervical cancer screening for HPV‐vaccinated women?

**DOI:** 10.1111/aogs.14978

**Published:** 2024-09-24

**Authors:** Jesper Bonde, Anne Hammer

**Affiliations:** ^1^ Department of Pathology, AHH‐Hvidovre Hospital Copenhagen University Hospital Hvidovre Denmark; ^2^ University Clinic for Gynecological HPV‐Related Disease, Department of Obstetrics and Gynecology Gødstrup Hospital Gødstrup Denmark; ^3^ Department of Clinical Medicine Aarhus University Aarhus Denmark

The HPV vaccine has been successful in reducing the incidence of cervical cancer and its precursors.^1^ Without a doubt or discussion. Thus, in 2018, the World Health Organization declared that cervical cancer could be eliminated within the next century (defined as an incidence rate below 4/100 000) if 90% of women receive the HPV vaccine, 70% undergo high‐performance screening at least twice, and 90% of women with cervical precancer receive adequate treatment.^2^, ^3^

Recently, a Danish study published in the *International Journal of Cancer* reported that cervical cancer incidence is down to 3 per 100 000 among women aged 20–29, suggesting the elimination of cervical cancer in this group of women.^4^ As a result of these findings, health economist, Professor Jakob Kjellberg, was quoted in Danish Broadcasting Corporation (DR) saying that screening of HPV‐vaccinated women is “overkill and a waste of money” and that discontinuation of screening in this group of women could save hundreds of millions (of Danish Crowns).^5^ The news piece was as short as it was unnuanced. Not a single healthcare professional working within the field was consulted for comments.

It is well known that a 3‐year cytology screening for HPV‐vaccinated women below 30 years as in Denmark and other Nordic countries has decreasing diagnostic value.^6^ In that context Professor Kjellberg has a point: A different approach is definitively needed, but discontinuation? Here are some reasons why discontinuation is jumping to conclusions.

In Denmark and Sweden, females have been immunized with the bi‐ or quadrivalent vaccine, protecting against HPV16 and HPV 18 until 2019. From 2019, the nonavalent vaccine has been in use. In Norway and Finland, the bivalent vaccine is still used in the HPV vaccination program, while the nonavalent vaccine is available via prescription. Although responsible for 70% of the cervical cancer cases,^7^, ^8^ the bivalent and quadrivalent HPV vaccines do not protect against the remaining 10 genotypes classified as oncogenic and constituting 30% of cervical cancers. This is important! The proportion of cervical cancers attributed to non‐HPV16/18 genotypes increases with age and constitute about half of all cervical cancers in older women.^9^ To cease screening among women immunized with the bivalent or quadrivalent HPV vaccine would be a disservice to those women. Also, age at the time of vaccination matters. Studies have shown optimal impact if the vaccine is given to HPV‐naïve persons, in effect, prior to sexual debut which in practical terms is before age 15/16 years. In contrast, there is limited impact of quadrivalent HPV vaccination on cancer rates in those aged 20 or above at the time of HPV vaccination.^1^ Thus, if screening recommendations are to be revised, it would be beneficial to link person‐level information on HPV vaccination status to the screening program, as this would enable targeted screening of those unvaccinated. This remains a technical gap.

And what about the HPV ecosystem? Whereas the HPV vaccines will result in fewer cervical cancers attributed to HPV genotypes included in the vaccines, the selective eradication of some HPV genotypes changes the viral ecosystem, a phenomenon called “unmasking.” Viral unmasking may occur when less common oncogenic HPV genotypes, previously overshadowed by more prevalent and aggressive oncogenic HPV genotypes such as HPV 16, are detected in screening, while clinical unmasking is when these types result in the development of precancer and cancer. In other words, HPV16/18‐associated cervical precancer and cancer is likely prevented by the HPV vaccine, but disease associated with non‐HPV16/18 genotypes may occur more often than previously reported, in effect decreasing the protection of the vaccine. In real life, unmasking will most likely affect the incidence of disease very little, but a key point is that the non‐vaccine, unmasked oncogenic HPV genotypes will be detected through screening.

But what about those unvaccinated, which is approx. 15%–20% of women in the Nordic countries? In the childhood program, parents choose whether their daughter is vaccinated or not. Later in life, the woman may decide to receive the HPV vaccine as a self‐paid vaccine, but the vaccine protection will be limited as described above. Yet, if screening is no longer in place for the vast majority of vaccinated women, will it be in place for those relatively few unvaccinated?

Many considerations should go into the question of cervical cancer screening of HPV‐vaccinated women. As always, it is about obtaining the right balance between benefits and harms. So, instead of a discussion on discontinuation or not, we should discuss optimizations, differentiation and timely healthcare service to all women, vaccinated or not. Should we delay initiation of cervical screening in those vaccinated? Should intervals be extended, for example, to 10 years following defined screening outcomes in vaccinated women? Can self‐collected samples be a more cost‐efficient approach to screen all women? Can biomarkers facilitate a better balance between sensitivity and specificity in vaccinated women undergoing screening? The nonavalent vaccine used in Denmark and Sweden from 2019 and onwards protects against the 7 oncogenic HPV types associated with 90% of cervical cancers, which just exaggerate the question on how to correctly conduct screening if any. Nevertheless, those vaccinated from 2019 and until now are years from reaching the screening age, so we have time.

But one thing stands. The topic is too important to be treated only in the context of a healthcare budget saving. The topic is also too important to be treated without professional qualification and reflection. How many women read the news piece in the context that, if HPV vaccinated, they do not need screening anymore?

In the Nordic countries, we have strong professional communities and healthcare institutions, and the topic of cervical cancer screening of vaccinated and unvaccinated women should be approached with respect, professionalism and due reflections on benefit and harms, and not just for a few pennies less or a few fast clicks on an otherwise easily forgotten news cycle.
